# The Study of External Dose Rate and Retained Body Activity of Patients Receiving ^131^I Therapy for Differentiated Thyroid Carcinoma

**DOI:** 10.3390/ijerph111010991

**Published:** 2014-10-21

**Authors:** Haiying Zhang, Ling Jiao, Songye Cui, Liang Wang, Jian Tan, Guizhi Zhang, Yajing He, Shuzhou Ruan, Saijun Fan, Wenyi Zhang

**Affiliations:** 1 Tianjin Key Laboratory of Molecular Nuclear Medicine, Institute Of Radiation Medicine, Chinese Academy Of Medical Sciences & Peking Union Medical College, Tianjin 300192, China; E-Mails: chsyzhy@163.com (H.Z.); ljiao.irm@163.com (L.J.); cuisongye@gmail.com (S.C.); wangliang2012ss@163.com (L.W.); ruanshuzhou@163.com (S.R.); fansaijun@irm-cams.ac.cn (S.F.); 2 Department of Nuclear Medicine, Tianjin Medical University General Hospital, Tianjin 300052, China; E-Mails: tanpost@163.com (J.T.); guizhi_zhang@163.com (G.Z.); tjzyyhyj@163.com (Y.H.)

**Keywords:** radiation safety, differentiated thyroid carcinoma, ^131^I, external dose rate, retained body activity

## Abstract

Radiation safety is an integral part of targeted radionuclide therapy. The aim of this work was to study the external dose rate and retained body activity as functions of time in differentiated thyroid carcinoma patients receiving ^131^I therapy. Seventy patients were stratified into two groups: the ablation group (A) and the follow-up group (FU). The patients’ external dose rate was measured, and simultaneously, their retained body radiation activity was monitored at various time points. The equations of the external dose rate and the retained body activity, described as a function of hours post administration, were fitted. Additionally, the release time for patients was calculated. The reduction in activity in the group receiving a second or subsequent treatment was more rapid than the group receiving only the initial treatment. Most important, an expeditious method was established to indirectly evaluate the retained body activity of patients by measuring the external dose rate with a portable radiation survey meter. By this method, the calculated external dose rate limits are 19.2, 8.85, 5.08 and 2.32 μSv·h^−1^ at 1, 1.5, 2 and 3 m, respectively, according to a patient’s released threshold level of retained body activity <400 MBq. This study is beneficial for radiation safety decision-making.

## 1. Introduction

Since the advent of nuclear medicine, radionuclides have been increasingly used in clinical diagnose and treatment [1,2,3]. Among them, ^131^I is often used for treating thyroid diseases, showing a remarkable curative effect, especially in thyroid carcinoma. The remnant tissue and metastasis of the differentiated thyroid carcinoma (DTC) have a certain capacity for taking up ^131^I, so the beta rays emitted by ^131^I can be used to destroy them, which is key to achieving treatment goals. This treatment reduces the risk of relapse and of metastasis after surgery for DTC and improves the survival rate. At present, ^131^I treatment has become an idle formality after surgery for DTC, with typical prescribed activities ranging from 1,850 to 7,400 MBq [4]. Nevertheless, patients become an unsealed source of radioactive contamination, which can easily cause external radiation exposure to nearby individuals due to the high administered activity and the high energy and strong penetrating power of gamma rays. Therefore, ^131^I has become the main radiation safety concern in the nuclear medicine department. Furthermore, the level of the external dose rate (EDR) and retained body activity (RBA) of the patient has become a criterion for releasing patients [4].

Both, the publication number 60 of the International Atomic Energy Agency (IAEA) and the Chinese national basic standards for ionizing radiation protection and radioactive source security put forward strict rules about radiation safety in radionuclide therapy [5]. The latter recommends that the annual dose an individual receives should not exceed 5 mSv. Thus, regulations are implemented to limit the radiation exposure of the medical staff, relatives and caregivers with whom patients may come into contact.

The regulations vary between countries, but they are usually based on the measurement of the EDRs or the RBAs of patients. In 2006, the IAEA recommended 1,200 MBq or 70 μSv h^－1^ at 1 m from patients as the directive level for releasing patients [6]. The guidance of the European Union (EU) states that, for releasing patients receiving ^131^I therapy, the RBA must be <400 MBq and the EDR at 1 m must be <20 μSv·h^−1 ^ [7]. According to China’s latest occupational health standards, patients receiving ^131^I therapy cannot be released until their RBA is reduced to 400 MBq.

Admittedly, maybe, the EDR and the RBA functions for the ^131^I administered to patients have been well characterized and widely used. However, some important issues still remain to be resolved. These include, first, the facts that prior studies were often concerned with only one of EDR or RBA [8,9,10] and that there was no function for describing both of them when obtained in an identical cohort of patients at the same time. Second, despite the existing work, the data involving the Eastern World population in this field are absent. It is essential to carry out this study in this population, because the data from population-based studies in the Western World countries are not representative of the Chinese populations, due to the big difference in diet, region, body type and race. Third, although a few of the previous studies roughly demonstrated that the EDR had a positive correlation with the RBA [11,12], no available function had yet been established for specifically describing the relationship between them. Hence, it is important to estimate RBA by EDR measurement. Fourth, the data from previous studies are imprecise, since not only were they obtained without the correction for the time attenuation of radiation activity, but also they failed to include some abnormal cases [12]. In addition, the measuring frequency in prior studies was somewhat loose [8,9]; in the present study, 10 time points were measured within 72 h, and such tighter measurements contributed to a better characterization of the time-dependent change in the EDR and BRA. Finally, the American Thyroid Association approximates the patient as a point source [13]. Nonetheless, the point source approximation may be excessively conservative.

The aim of this work was to: (1) study the EDR and RBA as a function of time for patients receiving ^131^I therapy for DTC; and (2) establish the relationship between the EDR and RBA. Through the use of these functions, the RBA of patients can be indirectly estimated by measuring the EDR with a portable radiation survey meter, which should expedite the decision of whether patients can be safely discharged.

## 2. Experimental Section

### 2.1. Patients’ Materials

This prospective study was approved by our institutional review board. Written formal consent was obtained from all of the patients following an explanation of the study. The EDR and RBA were measured in consecutive patients receiving ^131^I therapy for DTC. A total of 70 patients were recruited: 54 patients were females and 16 were males with age ranging from 24–68 years (mean 47 ± 12.3 years). This study population consisted of two groups, the ablation group (A) and the follow-up group (FU). The former included 27 patients receiving ^131^I therapy for the first time, to ablate the thyroid after surgery; the latter comprised 43 patients receiving second or subsequent ^131^I therapy for suspected residual, recurrent or metastatic disease. The administered activity was 3,605 ± 663 MBq (range 1,184–4,033 MBq) to group (A) and 3,669 ± 829 MBq (range 1,887–5,550 MBq) to group (FU). All patients were diagnosed with papillary thyroid carcinoma by pathology. All patients had been previously treated with total or near total thyroidectomy and interrupted thyroid hormone reposition for 4–6 week before receiving ^131^I therapy. All patients were prescribed a low-iodine diet before therapy. Pregnancy and diseases affecting micturition, such as kidney disease, were not diagnosed among any of the patients.

### 2.2. Measurement of the EDR

To reduce errors, all of the measurements in this work were performed by the same experimenter at the same time every day. The ^131^I administration started at 14:00 on the first day, in the hospital. In order to avoid disrupting the patients’ sleep time, the EDRs were measured at 1, 6, 18, 24, 30, 42, 48, 54, 66 and 72 h post ^131^I administration. The initial measurement was performed at 1 h after ^131^I administration, when no urinary excretion had occurred yet. The surveying was conducted in a special room, where the radiation level was consistent with the natural background. Every EDR measurement was accompanied by the measurement of the background EDR, and the net patient EDR was calculated. Three readings were taken for each measurement and were averaged. The EDRs were measured with a portable radiation survey meter with a NaI (T1) scintillator detector (RadEye PRD, Thermo Fisher Scientific Inc., Waltham, MA, USA), which was calibrated annually by the National Institute of Metrology of China. The distances were measured by a laser distance meter (Disto D2, Leica Geosystems AG, St. Gallen, Switzerland). During measurement, patients were required to sit in a chair and keep their upper bodies straight. The EDRs were measured at 1, 1.5, 2 and 3 m in front of the patients’ thyroid gland at a height of 1 m above the floor.

### 2.3. Estimation of the RBA

Patients were asked to empty their bladders before ^131^I administration. Urine was collected immediately after the EDR measurement and measured with specialized graduated vessels. The urine volume at every time point until 72 h was recorded.

A radioactivity calibrator (CRC-15R, Ramsey, NJ, USA), which was calibrated by National Institute of Metrology of China, was used to measure the activity in the urine. The urine was stirred before sampling to make the activity homogeneously distributed. A 1-mL volume of urine sample was used for measuring. After each measurement, the urine was poured out, and a new vessel was used. According to the measured activity and the urine volume, the activity in the urine was calculated at every time point. Subsequently, the retained ^131^I body activity was estimated, by subtracting the activity in the urine from the administered activity after correcting for time attenuation as follows:

(1) $${\text{RBA}}_{\text{m}}={\text{RBA}}_{\text{n}}{\text{e}}^{-\frac{0.693}{T_{p}}\text{t}}-{\text{RBA}}_{\text{mnu}}$$

Here, RBA_m_ and RBA_n_ are the RBA at m h and n h (m > n). RBA_mnu_ and t are the activity in the urine and the time span between m h and n h, respectively. *T_p_* is the physical half-life of ^131^I.

## 3. Results and Discussion

The radioactive isotope ^131^I was selected to treat patients for DTC, mainly due to the specific ability of thyroid tumor cells to take up this isotope ^131^I, which emits beta rays to kill thyroid cancer cells and eliminate the focus [14]. However, ^131^I also emits gamma rays, whose strong ionizing radiation effects can cause damage to nearby individuals. In addition, the treated patient could spread radioactive contamination and pollute the surroundings. Therefore, before discharging the patients, the EDR and BRA must be reduced to an acceptable level.

### 3.1. EDR

**Figure 1:**
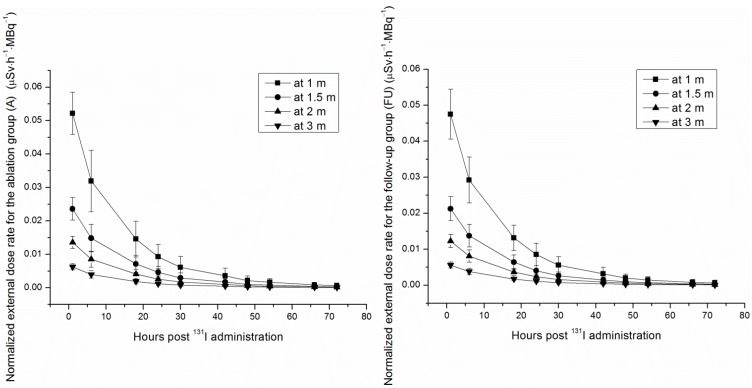
External dose rates at 1 m, 1.5 m, 2 m and 3 m plotted as a function of hours post ^131^I administration for the ablation group (A) and the follow-up group (FU).

The initial EDR obtained at 1 h was considered to be the baseline quantification with 100% of the activity administered at 0 h. In order to facilitate comparison, EDR was normalized to administered activity. The normalized EDR *vs.* time, which decays bi-exponentially in the two groups (A and FU) according to Equations (2)–(9), is shown in Figure 1:

(2) $${\text{EDR}}_{\text{A}1}=0.04477{\text{e}}^{-0.0638\text{t}}+0.01504{\text{e}}^{-0.396\text{t}}$$

(3) $${\text{EDR}}_{\text{A}1.5}=0.02179{\text{e}}^{-0.0637\text{t}}+0.0366{\text{e}}^{-2.4521\text{t}}$$

(4) $${\text{EDR}}_{\text{A}2}=0.01254{\text{e}}^{-0.064\text{t}}+0.02{\text{e}}^{-2.42\text{t}}$$

(5) $${\text{EDR}}_{\text{A}3}=0.00585{\text{e}}^{-0.0665\text{t}}+0.00983{\text{e}}^{-2.5992\text{t}}\;$$

(6) $${\text{EDR}}_{\text{FU}1}=0.03971{\text{e}}^{-0.0629\text{t}}+0.01412{\text{e}}^{-0.325\text{t}}\;$$

(7) $${\text{EDR}}_{\text{FU}1.5}=0.02024{\text{e}}^{-0.0652\text{t}}+0.00473{\text{e}}^{-0.7281\text{t}}$$

(8) $${\text{EDR}}_{\text{FU}2}=0.01056{\text{e}}^{-0.061\text{t}}+0.00292{\text{e}}^{-0.2192\text{t}}$$

(9) $${\text{EDR}}_{\text{FU}3}=0.00357{\text{e}}^{-0.054\text{t}}+0.00249{\text{e}}^{-0.1172\text{t}}$$

Here, EDR_A1_, EDR_A1.5_, EDR_A2_, EDR_A3_, EDR_FU1_, EDR_FU1.5_, EDR_FU2_ and EDR_FU3_ are the normalized EDRs at the 1, 1.5, 2 and 3 m distances of group (A) and group (FU). t is the time post ^131^I administration.

In this study, the EDRs of group (A) at 1, 6, 18, 24, 30, 42, 48, 54, 66 and 72 h at 1 m were 0.052 ± 0.006, 0.032 ± 0.009, 0.015 ± 0.005, 0.009 ± 0.003, 0.006 ± 0.003, 0.003 ± 0.002, 0.002 ± 0.001, 0.002 ± 0.001, 0.001 ± 0.001 and 0 ± 0 μSv h^﹣1^ MBq^﹣1^, respectively; the EDRs of group (FU) at 1, 6, 18, 24, 30, 42, 48, 54, 66 and 72 h at 1 m were 0.047 ± 0.007, 0.029 ± 0.006, 0.013 ± 0.003, 0.009 ± 0.003, 0.006 ± 0.002, 0.003 ± 0.002, 0.002 ± 0.001, 0.001 ± 0.001, 0.001 ± 0.001 and 0 ± 0 μSv·h^−1^·MBq^−1^, respectively. The results from similar studies are not always concordant. In a report by Barrington, S.F. *et al.* [3], in which 83 thyroid carcinoma patients from the U.K. were studied, the EDRs of the group similar to group (A) at 24, 48 and 72 h at 1 m were 0.019 ± 0.006, 0.009 ± 0.006 and 0.007 ± 0.007 μSv·h^−1^·MBq^−1^, respectively; the EDRs of the group similar to group (FU) at 24, 48 and 72 h at 1 m were 0.019 ± 0.02, 0.007 ± 0.006 and 0.003 ± 0.002 μSv·h^−1^·MBq^−1^, respectively. Thus, it can be seen that the EDRs decreased faster in the Chinese patients than in the U.K. patients. This is likely caused by varying races, body types, dietary habits, environmental conditions, *etc.* The effects of these factors on EDR clearance are not yet accounted for, however, warranting further research in the future.

A bi-exponential model was used to describe the relationship between EDR and elapsed time post ^131^I administration. Typically, the EDR decreased with time for the natural attenuation and urinary excretion. However, a particular patient’s EDR in group (A) increased through the 48–54-h time interval, but decreased as time went on through the other periods of time. The intake of sialagogues (e.g., lemon juice, sour candies, lemon candies) may cause this phenomenon. The mechanism is that the ingested sialagogues stimulate the secretion of the ^131^I from the salivary glands. This, in turn, causes a decrease of radioiodine uptake in the salivary glands. With thyroid radioactivity, the radioactivity in salivary glands affected the EDRs measured by external counting at varying distances from the patient’s neck [15]. Hence, it is likely that the patient had some sour candies (distributed by the doctor to protect the salivary glands) before the 48-h time point, and then, the iodine uptake of the salivary glands was reduced near the 48-h time point, but recovered during the 48–54-h time interval.

### 3.2. The Point Resource

**Figure 2:**
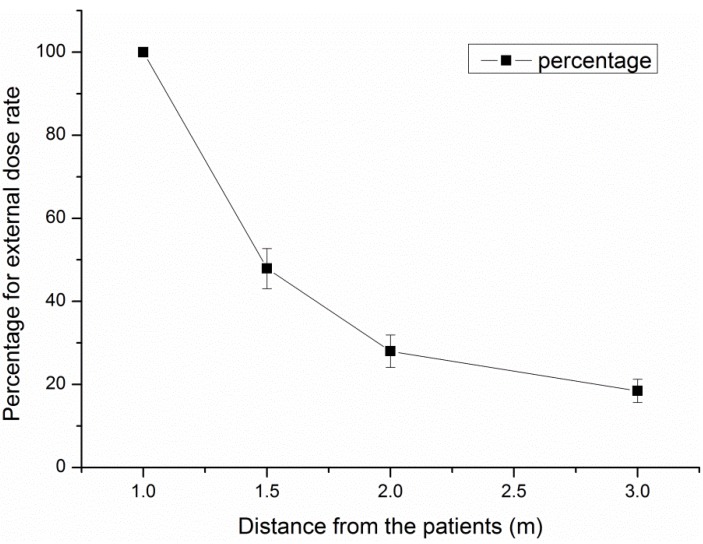
The percentage of external dose rate changes along with distance from the patients.

In calculations of the doses absorbed by patients receiving ^131^I therapy, the activity distribution in such patients is commonly assumed to be an unattenuated point source, and the dose to exposed individuals at a given distance is hence calculated using the inverse square law [16]; whereas the data in this study follow a different law, which is shown in Figure 2 and described in Equation (10):

(10) $$\text{EDR}={\text{EDR}}_{1}\times {\text{r}}^{-1.834}$$

Here, r is the distance from the patients. Songye Cui *et al.* [17] measured the EDR in 48 patients receiving ^131^I therapy at 0.3, 0.6, 1 and 2 m and found that the EDRs varied with distance; such a result is consistent with the results derived from Equation (10) in this study.

A reason that may account for this discrepancy is that ^131^I may not have distributed over a concentrated location in the patients’ body; thus, it could not be treated as a point radiation source. Actually, a previous study indicated that due to the nature of the ^131^I activity distribution in thyroid cancer therapy, the patient can be modeled as a line source [18].

### 3.3. RBA

**Figure 3:**
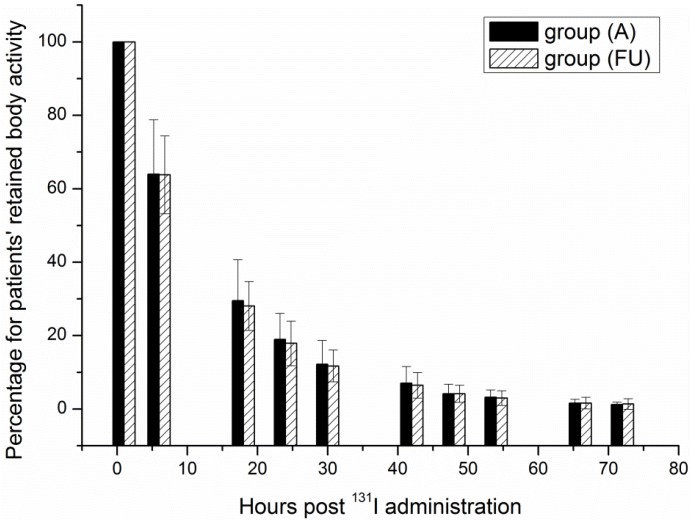
Retained body activity plotted as a function of hours post ^131^I administration.

After radioiodine treatment, the urinary system is generally considered the major route of ^131^I excretion, although there are other excretion routes, such as exhaled breath, perspiration and saliva [4]. Hence, BRA was calculated by measuring the activity in the urine and subtracting it from the administered activity after correcting for time attenuation. The results, illustrated in Figure 3, revealed that even though the administered activities were higher in group (FU) than in group (A), the group (FU) cleared the activities more rapidly. The most obvious reason for such a result is that the thyroid tissue of the group (FU) was most likely almost completely ablated, so the reduction in activity in this group was more rapid than in group (A). The excretion of ^131^I was fast during the first 24 h and became increasingly slow afterwards. Nevertheless, it was obvious that most of the activity was eliminated within the first 72 h.

The activity decreased bi-exponentially in the two groups as follows:

(11) $${\text{RBA}}_{\text{A}}={\text{RBA}}_{0}\left(0.84{\text{e}}^{-0.0617\text{t}}+0.16{\text{e}}^{-0.1584\text{t}}\;\right)$$

(12) $${\text{RBA}}_{\text{FU}}={\text{RBA}}_{0}\left(0.69{\text{e}}^{-0.0877\text{t}}+0.31{\text{e}}^{-0.0473\text{t}}\;\right)$$

Here, RBA_A_ and RBA_FU_ are the RBA in patients of group (A) and group (FU). t is the time post ^131^I administration, and RBA_0_ is the administered activity.

The results of this study indicated that the RBA rapidly decreased within the first 24 h, and there was not much left at 72 h. Therefore, after being released from the hospital, the patients’ RBA becomes sufficiently low in a much short time, thereby reducing the risk of exposure to the general public, which concurs with previous findings [19]. For typical activities administered to the patients of 1,850 MBq, 3,700 MBq and 5,550 MBq, the release time for patients, listed in Table 1, refers to the point in time when the selected cutoff level of 400 MBq is reached. Such a rapid clearance of RBA means that the therapy could be administered at home under suitable domestic circumstances. After all, the concentration of ^131^I in domestic drainage systems should not pose a significant risk [20].

**Table 1:** Release time for patients (h).

Group	Activity Administered to Patients (MBq)		
	1850	3700	5550
group (A)	22.3	33.4	39.9
group (FU)	21.5	32.1	38.8

The application of the time attenuation correction for radiation activity means that some activities were eliminated via routes other than urinary excretion, and the results should thus be more precise than those from studies, such as in Demir *et al.* [12], in which no correction was made for time attenuation.

### 3.4. The Relationship between EDR and RBA

**Figure 4:**
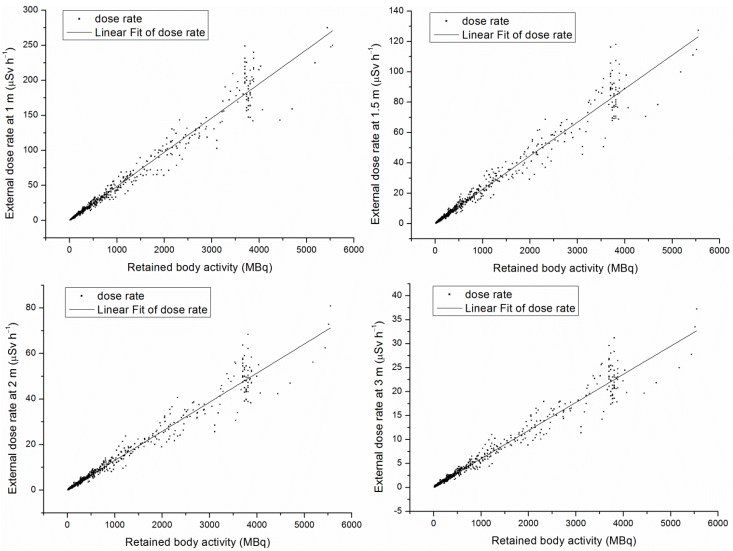
Retained body activity corresponding external dose rate.

Figure 4 shows a significant positive correlation between EDR at a given distance and the RBA calculated in this study. This results in:

(13) $${\text{EDR}}_{1}=0.048\text{RBA}$$

(14) $${\text{EDR}}_{1.5}=0.02212\text{RBA}$$

(15) $${\text{EDR}}_{2}=0.0127\text{RBA}$$

(16) $${\text{EDR}}_{3}=0.0058\text{RBA}$$

To confirm the release time, the BRA was routinely calculated in two ways: by measuring the activity in the collected patients’ urine every day and by detecting it though a whole body counter. The former work was burdensome and exposed collectors to a substantial amount of radiation, which could increase the risk of cancer over time; while the latter work was often considerably more expensive.

The measurement of the EDR around patients receiving ^131^I therapy is one of the established methods of estimation of RBA. Thomas *et al.* [21] compared three methods of evaluating RBA, which consisted of: (1) urine assay; (2) prediction based on a pre-therapy diagnostic workup method using 74 MBq; and (3) EDR measurements. They concluded that the direct EDR method was an accurate, reliable and safe method of monitoring the patient’s ^131^I RBA.

The present study ensured the accuracy of the direct EDR method by establishing a relationship between EDR and RBA with Equations (13)–(16) through actual measurements. With the equations, the estimation of the patients’ BRA was expedited by quickly measuring the EDR at a given distance with a portable radiation survey meter. In order to assess various situations, measurements were made at four different distances. In addition, this method proved to be simpler and will result in less radiation exposure than with the urine assay method. Also noteworthy, compared to the whole-body counting method, this method is superior cost wise. When Equation (13) was applied, the EDR at 1 m per MBq was 0.048; such a result was higher than that reported by Demir M *et al.* [12]. In their study, the EDRs at 1 m per MBq, taking the average of patients, was calculated as 0.042 ± 0.01. According to China’s activity limit level of 400 MBq, the authors recommend that the limit level of <19.2 μSv·h^−1^ at 1 m should be set for China when the RBA calculated by Equation (13) is 400 MBq. This proportional relationship between EDR and RBA was nearly consistent with the guidance of the IAEA [6] and the European Union [7]. Moreover, the EDR limit levels, as calculated by Equations (14)–(16) according to the limit level of 400 MBq, are 8.85, 5.08 and 2.32 μSv h^﹣1 ^at 1.5, 2 and 3 m, respectively.

As shown in Figure 4, the EDR sharply rose and fell between 3000 and 5550 MBq, which were mainly the administered activities. This effect may be due to two reasons: (1) the time for the ^131^I to be absorbed from the stomach to the blood; and (2) the retention of iodine in the urine while in the bladder.

### 3.5. Normalized Cumulative Dose

The International Commission on Radiological Protection (ICRP) insists that medical staff, relatives and caregivers receive the biggest dose while patients are undergoing ^131^I therapy [4]. Dose estimates should be used to evaluate the appropriate restrictions related to personnel’s behavior to limit the received dose. The cumulative dose received by a nearby individual depends mainly on the time, distance and administered activity.

The systematically developed database in Table 2 can be used for estimating the individuals’ normalized cumulative dose at different distances. This database assumed that the 1-m distance instructed by doctors is the minimal safe distance and that 3 m is the maximum distance between an individual and a patient in a ward. Through the database, the radiation hazard to an individual at each period of time was clear. The normalized cumulative dose of a random period of time can be obtained according to the follow equation:

**Table 2:** Normalized cumulative dose around patients in each period of time post ^131^I administration (μSv·MBq^−1^).

Group	Distance	Period of Time Post ^131^I Administration											
		0–6 h	6–12 h	12–18 h	18–24 h	24–30 h	30–36 h	36–42 h	42–48 h	48–54 h	54–60 h	60–66 h	66–72 h
group (A)	1 m	0.25763	0.15540	0.10409	0.07081	0.04827	0.03292	0.02245	0.01531	0.01044	0.00712	0.00485	0.00331
	1.5 m	0.12358	0.07414	0.05059	0.03452	0.02356	0.01607	0.01097	0.00748	0.00511	0.00348	0.00238	0.00162
	2 m	0.07074	0.04256	0.02899	0.01974	0.01345	0.00916	0.00624	0.00425	0.00289	0.00197	0.00134	0.00091
	3 m	0.03272	0.01942	0.01303	0.00874	0.00587	0.00394	0.00264	0.00177	0.00119	0.00080	0.00054	0.00036
group (FU)	1 m	0.23573	0.14137	0.09405	0.06408	0.04387	0.03007	0.02062	0.01413	0.00969	0.00665	0.00456	0.00312
	1.5 m	0.10692	0.06805	0.04596	0.03108	0.02102	0.01421	0.00961	0.00650	0.00440	0.00297	0.00201	0.00136
	2 m	0.06280	0.03941	0.02622	0.01789	0.01232	0.00852	0.00591	0.00409	0.00284	0.00197	0.00137	0.00095
	3 m	0.02903	0.01854	0.01220	0.00822	0.00565	0.00394	0.00278	0.00197	0.00141	0.00101	0.00073	0.00052

(17) $$\text{H′}=\int_{{\text{t}}_{1}}^{{\text{t}}_{2}}\text{EDR}\left(\text{t}\right)\text{dt}$$

Here, H’ is the cumulative dose, t_1_ and t_2_ are the starting and ending time. EDR(t) is the one of Equations (2)–(9).

In a study by Willegaignon *et al.* [10], after being normalized to administered activity, the cumulative dose at 1 m in front of the patients was 0.56756 ± 0.08108, 0.16216 ± 0.08108 and 0.05675 ± 0.04324 μSv·MBq^−1^ through 0–24 h, 24–48 h and 48–72 h. According to Table 2, the normalized cumulative dose at 1 m in front of patients was 0.58793, 0.11895, 0.02572 in group (A), and 0.53528, 0.10869, 0.02402 in group (FU), through the same time intervals. Therefore, the normalized cumulative dose decreased faster around the Chinese patients than around the Brazilian patients.

### 3.6. Home Care Precautions

After being released from the hospital, patients’ EDR and RBA diminished to quite low levels, and small effects contributed to the cumulative radiation injuries over time, which was in accord with the principle of reducing radiation exposure to levels that are as low as reasonably achievable (ALARA). However, some home care precautions were necessary to limit the radiation exposure to family members. Specific practice recommendations proposed by the American Thyroid Association [13] include the following:

As for sleeping arrangements, patients should sleep in a separate bed at least six feet away from adults throughout the nighttime restricted period (one day for 1850, one day for 3700, two days for 5550 and four days for 7400 MBq administered); however, sleeping in a separate bedroom or area would be safest. For pregnant women, infants and children, a distance >6 feet away from the patients should be kept for the recommended restricted time (six days for 1850, 13 days for 3700, 18 days for 5550 and 21 days for 7400 MBq administered).

In addition, the following precautions regarding body wastes should be emphasized during the restricted periods. Patients should take a shower every day and not have intimate contact, such as kissing and sexual intercourse. Patients should clear the mouthpiece after using a phone shared with others. After using the toilet, the seat should be cleaned using wipes, the toilet and wipes flushed and the hands washed. Anything from the patients’ body should be flushed down the toilet, and things that cannot be flushed, such as menstrual pads, bandages, paper towels, spoons and forks, should be put in the appropriate specified plastic trash bag. After brushing the teeth, it is important for patients to flush the washing tank and wash hands to wash away the spit. Toothbrush, razor, face cloth, towel, food or drinks, spoons, forks, glasses and dishes are prohibited to share. Patients’ underwear, pajamas, sheets and any clothes that contain sweat, blood or urine should be washed regularly. Gloves used for clearing vomit, blood, urine or stool should be disposed of after each use in the specified plastic trash bags. The specified plastic trash bags should be placed in a separate area and far from children and animals. The bag may be discarded as general trash after 80 days.

## 4. Conclusions

By actual measurements, this work demonstrated that several bi-exponential models accurately described ^131^I-iodide kinetics up to 72 h post ^131^I administration to patients receiving ^131^I therapy, establishing functions that prove to be useful in estimating the RBA by measuring EDR. This allowed the systematic development of a database that provides experiment-based clinical practice guidelines to reduce radiation exposure. Considering the above mentioned facts, to hold the radiation dose to the nearby individuals as low as reasonably achievable, they and the patients should be properly educated in this aspect, and then, the dose delivered to them by patients would be within the permissible limits.
